# Chemistry and Biological Activities of the Marine Sponges of the Genera *Mycale* (*Arenochalina*), *Biemna* and *Clathria*

**DOI:** 10.3390/md16060214

**Published:** 2018-06-18

**Authors:** Amr El-Demerdash, Mohamed A. Tammam, Atanas G. Atanasov, John N. A. Hooper, Ali Al-Mourabit, Anake Kijjoa

**Affiliations:** 1 Muséum National d’Histoire Naturelle, Molécules de Communication et Adaptation des Micro-organismes, Sorbonne Universités, UMR 7245 CNRS/MNHN, CP 54, 57 Rue Cuvier, 75005 Paris, France; 2 Organic Chemistry Division, Chemistry Department, Faculty of Science, Mansoura University, Mansoura 35516, Egypt; 3 Department of Pharmacognosy and Chemistry of Natural products, Faculty of Pharmacy, National and Kapodistrian University of Athens, Panepistimiopolis Zografou, Athens 15771, Greece; mat01@fayoum.edu.eg; 4 Department of Biochemistry, Faculty of Agriculture, Fayoum University, 63514 Fayoum, Egypt; 5 Department of Pharmacognosy, University of Vienna, 1090 Vienna, Austria; atanas.atanasov@univie.ac.at; 6 Institute of Genetics and Animal Breeding of the Polish Academy of Sciences, 05-552 Jastrzebiec, Poland; 7 Queensland Museum, Biodiversity & Geosciences Program, P.O. Box 3300, South Brisbane BC, Queensland 4101, Australia; john.hooper@qm.qld.gov.au; 8 ICSN—Institut de Chimie des Substances Naturelles, CNRS UPR 2301, University of Paris-Saclay, 1, Avenue de la Terrasse, 91198 Gif-Sur-Yvette, France; Ali.ALMOURABIT@cnrs.fr; 9 ICBAS—Instituto de Ciências Biomédicas Abel Salazar & CIIMAR, Universidade do Porto, Rua de Jorge Viterbo Ferreira, 228, 4050-313 Porto, Portugal

**Keywords:** marine sponges, Poecilosclerida, Biemnida, *Mycale* (*Arenochalina*), *Biemna*, *Clathria*, crambescidins, batzelladines, guanidine alkaloids, pteridine alkaloids, terpenoids, thiopepetides, macrolides, polyketides, indole alkaloids, pyrrole-containing alkaloids, nucleotides, terpenoids, steroids, fatty acids

## Abstract

Over the past seven decades, particularly since the discovery of the first marine-derived nucleosides, spongothymidine and spongouridine, from the Caribbean sponge *Cryptotethya crypta* in the early 1950s, marine natural products have emerged as unique, renewable and yet under-investigated pools for discovery of new drug leads with distinct structural features, and myriad interesting biological activities. Marine sponges are the most primitive and simplest multicellular animals, with approximately 8900 known described species, although more than 15,000 species are thought to exist worldwide today. These marine organisms potentially represent the richest pipeline for novel drug leads. *Mycale* (*Arenochalina*) and *Clathria* are recognized marine sponge genera belonging to the order Poecilosclerida, whereas *Biemna* was more recently reclassified, based on molecular genetics, as a new order Biemnida. Together, these sponge genera contribute to the production of physiologically active molecular entities with diverse structural features and a wide range of medicinal and therapeutic potentialities. In this review, we provide a comprehensive insight and up-to-date literature survey over the period of 1976–2018, focusing on the chemistry of the isolated compounds from members of these three genera, as well as their biological and pharmacological activities, whenever available.

## 1. Introduction

Current medical risks, including diabetes, chronic pains, hepatitis, hypertension, microbial infection, together with the emergence of multidrug-resistant microbes and different types of carcinoma, have motivated and encouraged scientists to search for new bioactive compounds with novel modes of action [1]. Naturally occurring compounds derived from plants, marine invertebrates and microorganisms have provided important platforms and ideal validated starting materials for drug development and manufacturing [2]. Marine natural products represent a potent, promising and valuable source of supply for new chemical entities possessing unprecedented and novel mechanisms of action [2,3,4,5,6,7]. At present, marine-derived compounds or derivatives thereof have contributed to seven approved drugs for the market: cytarabine (Cytosar-U^®^, Depocyst^®^, approved by FDA in 1969 for cancer treatment), vidarabine (Vira-A^®^, approved by FDA in 1976 as antiviral), ziconotide (Prialt^®^, approved by FDA in in 2004 as analgesic for treatment of severe chronic pain), trabectedin (Yondelis^®^, ET-743, approved in the EU in 2007 as an anticancer), eribulin mesylate (Halaven^®^, approved by FDA in 2010 and by Heath Canada in 2011 for metastatic breast cancer treatment), brentuximab vidotin (Adcetris^®^, approved by FDA in 2011 for Hodgkin’s lymphoma cells, and in 2017 for cutaneous T-cell lymphoma) and omega-3 acid ethyl esters (Lovaza^®^, approved by FDA in 2004 for lowering blood triglyceride levels in adults with severe hypertriglyceridemia) [8,9]. Moreover, twelve marine natural products are being under exploration in different phases of clinical trials [8], and a number of them are in the preclinical pipeline. Despite being the most basal of metazoan animal phyla, marine sponges (Porifera) greatly contribute as prolific suppliers of potentially valuable novel compounds to the clinical pipeline, with almost 47% of all reported bioactive compounds from the marine environment. Several relevant reports have shown that almost 62.5% (i.e., 10 out of 16) of clinically approved medicines, or those in ongoing advanced clinical phases, are derived from marine invertebrates, including marine sponges [10,11]. Marine sponges of the genera *Mycale* (*Arenochalina*) (family Mycalidae), *Clathria* (family Microcionidae), and *Biemna* (family Desmacellidae) include diverse sponge species belonging to the orders Poecilosclerida and Biemnida. They are rich producers of diverse and physiologically active secondary metabolites [12,13] with a wide range of biological activities, including cytotoxic, antimalarial [14,15], anti-HIV [16], anti-inflammatory [17,18], enzyme inhibitors [19], antifungal and antibacterial properties [20,21]. Some of these compounds are chemotaxonomic markers, particularly for some Poecilosclerida marine sponges of the genera *Batzella*, *Crambe* and *Monanchora* [22]. The World Porifera Database [23] lists 14 valid species of *Mycale* (*Arenochalina*), 55 of *Biemna,* and 381 of *Clathria*. To the best of our knowledge, chemical investigations have previously been carried out only on nineteen species of the genus *Mycale* (*Arenochalina*), i.e., *Mycale* (*Arenochalina*) *mirabilis* and *Mycale* (*Arenochalina*) sp., *M. rotalis*, *M*. aff. *graveleyi*, *M*. *laxxissima*, *M. izuensis*, *M. fibrexilis*, *M. ancorina*, *M*. (carmia) cf. *spongiosa*, *M. adhaerens*, *M. magellanica*, *M. hentscheli*, *M. micracanthoxea*, *M. tenuispiculata*, *M. cecilia*, *M. laevis*, *M. lissochela* and *M. plumos*. For the genus *Biemna*, only four species, including *Biemna laboutei*, *Biemna* sp., *B. ehrenbergi*, and *B. fortis,* were chemically studied, while eleven species of the genus *Clathria,* i.e., *Clathria hirsuta, C. gombawuiensis, C. cervicornis, C. compressa, C. araiosa, Clathia.* sp., *C. calla, C. reinwardtii, C. lissosclera, C. basilana, C. strepsitoxa* and *C. pyramida* were chemically investigated (Table 1). Due to our interest in the marine sponges of the order Poecilosclerida [22,24,25,26], we have reviewed the literature reporting the isolation of secondary metabolites from these three marine sponge genera, covering the period of 1976–2018. This up-to-date review focuses mainly on the chemistry of the isolated metabolites, although their biological and pharmacological properties are also discussed when they are available.

## 2. Chemistry and Biological Activities of the Secondary Metabolites Isolated from the Marine Sponges of the Genera *Mycale* (*Arenochalina*)*, Biemna* and *Clathria*

In this section, we provide insights into the chemical classes and biological activities of the marine sponge-derived secondary metabolites obtained from these three genera. For convenience, the isolated compounds are divided into fourteen major groups, according to their skeleton as well as their biosynthetic origins. Additionally, their biological potentialities are also discussed whenever applicable.

### 2.1. Guanidine-Containing Alkaloids

**Figure 1:**
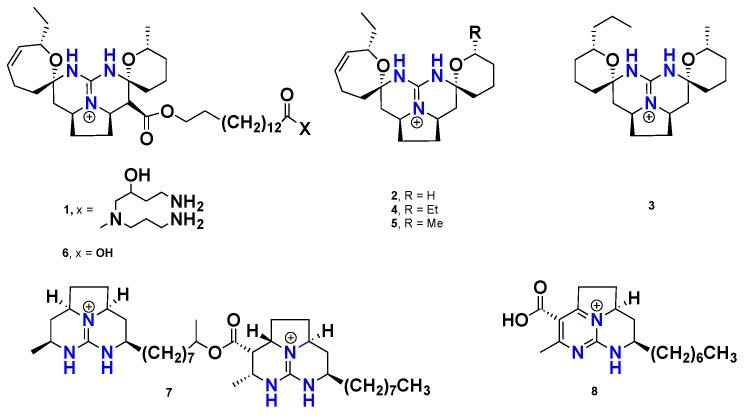
Chemical structures of **1**–**8**.

Crambescidins, batzelladines, mirabilins and ptilocaulins are definite groups of marine cyclic guanidine-containing alkaloids that display potent biological activities, such as cytotoxic, antiviral, antifungal and anti-HIV-1 gp 120-human. These compounds were isolated from various marine sponge genera, like *Batzella*, *Crambe*, *Monanchora* and *Ptilocaulis*, and are chemotaxonomic markers for the marine sponges belonging to the orders Poecilosclerida and Axinellida [22,27]. Crambescidin 800 (**1**), a pentacyclic guanidine alkaloid, was isolated from the marine sponge *Clathria* (*Thalysias*) *cervicornis*, and was found to display potent antimicrobial activity against *Acinetobacter baumannii, Klebsiella pneumoniae* and *Pseudomonas aeruginosa*, with MIC values of 2, 1 and 1 μg/mL, respectively [21]. Recently, three new crambescidin-type alkaloids, including crambescidin 345 (**2**), crambescidin 361 (**3**) and crambescidin 373 (**4**), along with the known congeners **1**, crambescidin 359 (**5**) and crambescidin 657 (**6**) (Figure 1), were isolated from the Indonesian marine sponge *C. bulbotoxa*. Interestingly, **3** was reported as a new crambescidin congener which possesses two identical saturated spiroaminal six-membered ring on both sides, which is considered to be rare within the crambescidin family. Additionally, **3** bears a propyl group as an alkyl substituent of the left-sided tetrahydropyran moiety. Compounds **2**–**5**, possessing only the pentacyclic guanidinium core (*vessel*), exhibited moderate cytotoxicity against the A431 cancer cell line with IC_50_ values of 7.0, 2.5, 0.94 and 3.1 μg/mL, respectively. However, **1** and **6**, featuring both the *vessel* and the long-chain ω-hydroxy fatty acid (*anchor*) motifs, displayed significant cytotoxicity with IC_50_ values of 48 and 12 nM, respectively. Such variation in cytotoxicity highlighted the importance of the spermidine part, which could act as a spacer linking two sites of interaction [24]. Furthermore, **2**–**4** demonstrated a strong anti-oomycete activity against the plant pathogenic fungus *Phytophthora capsici* with a minimum inhibitory dose (MID) of 50 μg/disk, while **1** and **6** showed a weak activity with MID 100 mg/disk or even higher [28]. Two batzelladine derivatives, norbatzelladine L (**7**) and clathriadic acid (**8**) (Figure 1), were isolated from the Caribbean marine sponge *C.* (*Microciona*) *calla*. Compound **7** exhibited potent cytotoxicity against a variety of cancer cell lines, including breast cancer (MDA-MB-231), non-small cell lung cancer (A549) and colon cancer (HT29), with GI_50_ = 0.7, 1.1 and 1.2 03BCg/mL, respectively, whereas **8** showed a weak antitumor activity with GI_50_ = 13.5, >30 and >30 μM, respectively. Moreover, **7** displayed stronger (IC_50_ = 0.4 μg/mL) antimalarial activity than **8** (IC_50_ = 2.3 μg/mL) [29].

**Figure 2:**
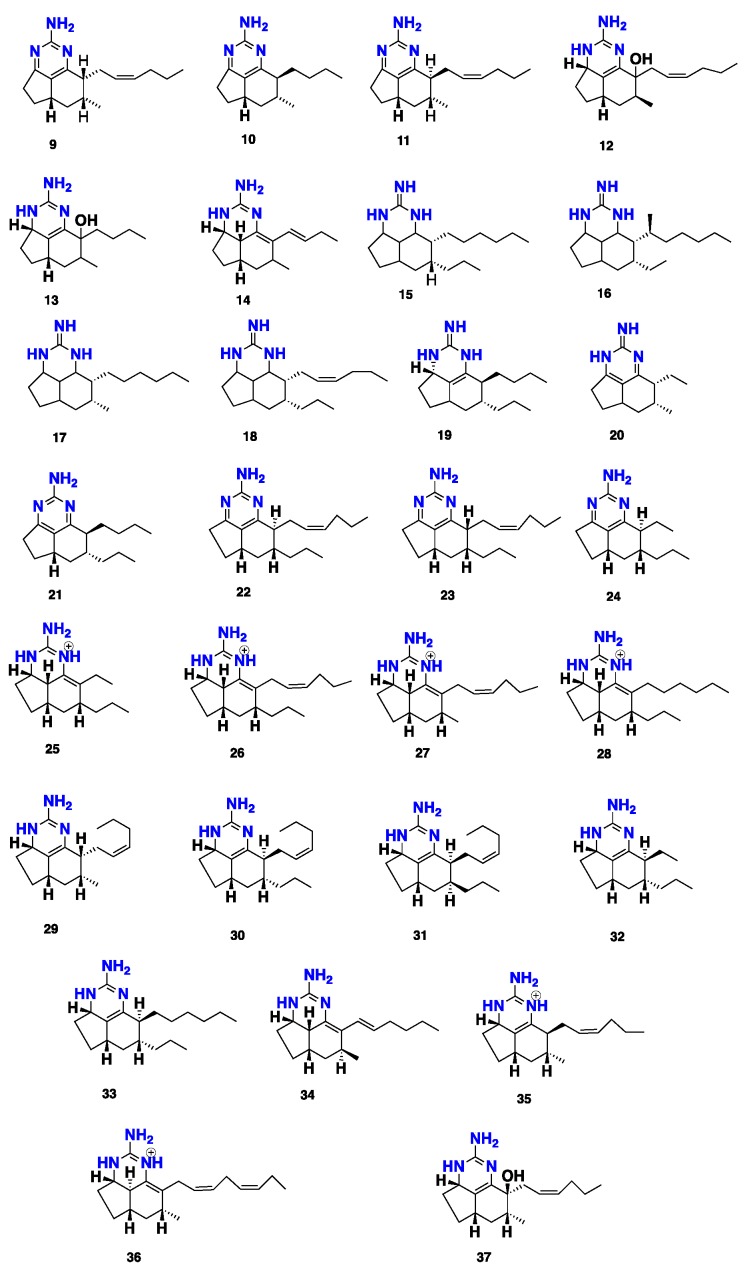
Chemical structures of **9**–**37**.

Six tricyclic guanidine alkaloids, mirabilins A–F (**9**–**14**), were isolated from a Southern Australian marine sponge *Mycale* (*Arenochalina*) *mirabilis* [30]. Later on, seven further cytotoxic tricyclic guanidine alkaloids, netamines A–G (**15**–**21**), were reported from a Madagascar marine sponge *Biemna laboutei.* These compounds showed an in vitro cytotoxic activity against three human cancer cell lines, i.e., NSCL (A549), colon (HT29), and breast (MDA-MB-231). While netamine C (**17**) showed GI_50_ values of 4.3, 2.4 and 2.6 μg/mL, respectively, netamine D (**18**) exhibited slightly higher GI_50_ values of 6.6, 5.3 and 6.3 μg/mL against these cancer cell lines [31]. An additional seven tricyclic alkaloids, netamines H–N (**22**–**28**), along with the known congeners netamine G (**21**) and mirabilins A (**9**), C (**11**) and F (**14**), were isolated from the same marine sponge. These compounds displayed cytotoxic and antimalarial activities. Netamine M (**27**) exhibited cytotoxicity against KB cancer cell line with the IC_50_ in a micromolar range whereas netamine K (**25**) showed antiplasmodial activity against *Plasmodium falciparum* with the IC_50_ value of 2.4 μg/mL [14]. Another five antimalarial tricyclic guanidine alkaloids, netamines O–S (**29**–**33**), were also isolated, together with the previously reported netamine E (**19**), from *B. laboutei*. Netamines O–Q (**29**–**31**) showed a promising in vitro antimalarial activity against *P. falciparum* with IC_50_ values of 16.99 ± 4.12, 32.62 ± 3.44, and 8.37 ± 1.35 μg/mL, respectively. Moreover, these compounds also exhibited cytotoxic activity against the KB cancer cell line in the range of 10^−5^ M [15]. A tricyclic guanidine alkaloid, mirabilin G (**34**), isolated from the Australian sponge *Clathria* sp., displayed a moderate antibacterial activity against Gram-negative bacterial strains, including *Escherichia coli* and *Serratia marcescens*, as well as antifungal activity against *Saccharomyces cerevisiae* [32]. Further chemical investigation of the marine sponge *Clathria* sp., collected from South Australia, resulted in the isolation of mirabilins C (**11**), F (**14**) and G (**34**), along with three new congeners, namely mirabilins H–J (**35**–**37**). Compounds **11**, **14**, **34**–**37** displayed no cytotoxicity against neuroblastoma (SH-SY5Y), gastric (AGS), colorectal (HT29) and intestinal (Intestine-407) cancer cell lines, with LD_50_ > 30 μg/mL [33] (Figure 2).

**Figure 3:**
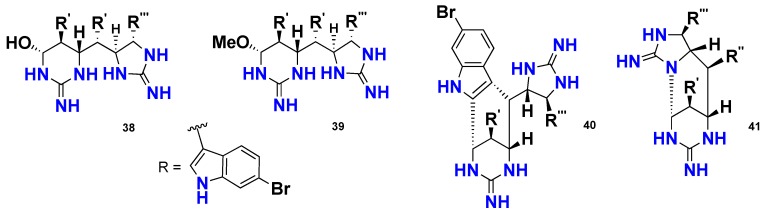
Chemical structures of **38**–**41**.

Another interesting group of marine cyclic guanidine alkaloids comprises those containing a bromoindole moiety*.* The *tris*-bromoindole cyclic guanidine alkaloids**,** araiosamine A–D (**38**–**41**) (Figure 3), were isolated from the marine sponge *Clathria* (*Thalysias*) *araiosa,* collected from Vanuatu. These compounds originated from an unusual mode of linear polymerization of tryptamine units involving a C–C bond formation. Compounds **38**–**41** were evaluated for their antimicrobial activity; however, none of them displayed significant antibacterial activity against *S. aureus* or anti-HIV activity [34].

### 2.2. Pyridoacridine, Pteridine, Tetrahydroquinolizine and N-methylpyrrolidone Alkaloids

**Figure 4:**
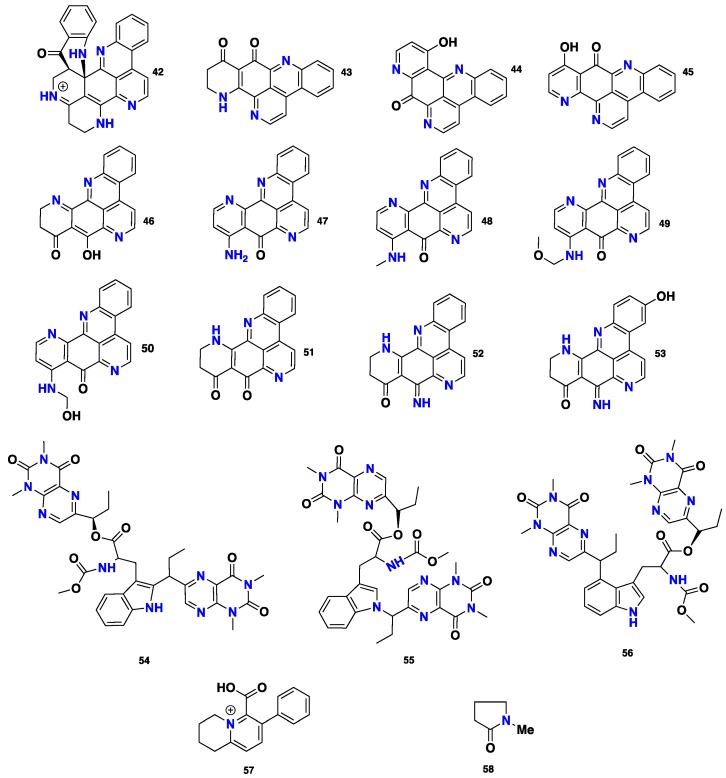
Chemical structures of **42**–**58.**

Pyridoacridine alkaloids are a unique group of marine-derived metabolites and are one of the largest marine alkaloid families. Chemically, they feature a common tetracyclic hetero-aromatic parent-11*H*-pyrido[4,3,2*nm*] acridine or 4*H*-pyrido[2,3,4-*kl*] acridone [35,36]. Among the three marine sponge genera, pyridoacridine alkaloids were exclusively isolated from *Biemna* species. Biemnadin (**42**), 8, 9-dihydro-11-hydroxyascididemin (**43**), 8-hydroxyisocystodamine (**44**) and 9-hydroxyisocystodamine (**45**) (Figure 4), were reported from the Okinawan *Biemna* sp. Compounds **42** and **43** displayed a significant in vitro cytotoxicity against two tumor cell lines: human epidermoid carcinoma KB (with IC_50_ values of 1.73 and 0.209 μg/mL, respectively) and murine lymphoma L1210 (with IC_50_ values of 4.29 and 0.675 μg/mL, respectively) [37]. Moreover, labuanine A (**46**) was isolated, along with three previously described congeners, i.e., **42**, **45** and isocystodamine (**47**) (Figure 4), from the Indonesian sponge *B. fortis.* All of these compounds induced multipolar neuritogenesis in more than 50% of Neuro 2A murine neuroblastoma cells at concentrations of 0.03–3 μM. Interestingly, **47** not only displayed the strongest neuritogenic activity but also activated an increase of the acetylcholinesterase level [38]. Matsunaga’s group [39] described the isolation of *N*-methylisocystodamine (**48**) and methoxymethylisocystodamine (**49**) (Figure 4), together with **47**, from the marine sponge *Biemna* sp*.,* collected at Oshima-Shinsone, Southern Japan. Both **48** and **49** were found to activate the erythroid differentiation of human leukemia K562 cells, with an ED_50_ value of 5 nM [39]. Later on, the same group [40] further isolated *N*-hydroxymethylisocystodamine (**50**) and neolabuaninen A (**51**), together with the previously reported congeners ecionines A (**52**) and B (**53**), **42**, **45** and **47** (Figure 4), from the same sponge. These compounds displayed cytotoxicity and activated differentiation of K562 leukaemia cells into erythrocytes at a concentration of 5 μg/mL. Furthermore, **47** and **50** were the most active in inducing neuronal differentiation when compared to **42**, **45** and **51**. Interestingly, while **51** and **52** lowered this activity, **42**, **47** and **53** showed no notable activity [40]. Another interesting group of marine-derived alkaloids are the pteridines, which represent a widely distributed family of naturally occurring alkaloids. Chemically, pteridine nucleus is composed of a pyrimidine ring fused with a pyrazine ring. Examples of this group are pseudoanchnazines A–C (**54**–**56**) (Figure 4), which were isolated from the marine sponge *Clathria* sp., collected near the coast of Rio Negro, Argentina. Compound **54** showed a moderate inhibition against *E. coli* at 50 μg/disk [41]. Additionally, Sperry and Crews described isolation of a new tetrahydroquinolizinium ion, clathryimine A (57), which produced a decarboxylated derivative clathryimine B upon heating in CDCl_3_ (Figure 4), from the Indo-Pacific marine sponge *C. basilana*, collected in Indonesia [42]. Radhika et al. [43] reported the isolation of *N*-methylpyrrolidone (**58**) (Figure 4) from *C. frondifera,* collected from the East coast of India.

### 2.3. Monoindole Alkaloids

**Figure 5:**
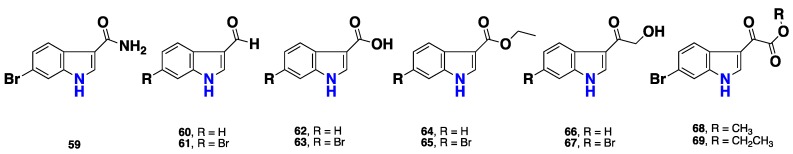
Chemical structures of **59**–**69**.

Wang et al. [44] reported the isolation of eleven brominated indole alkaloids, **59**–**69** (Figure 5), from the marine sponge *M. fibrexilis.* Since monoindole alkaloids were less common for this sponge family, the authors proposed that they could be specific for this species.

### 2.4. Pyrrole-Containing Alkaloids

**Figure 6:**
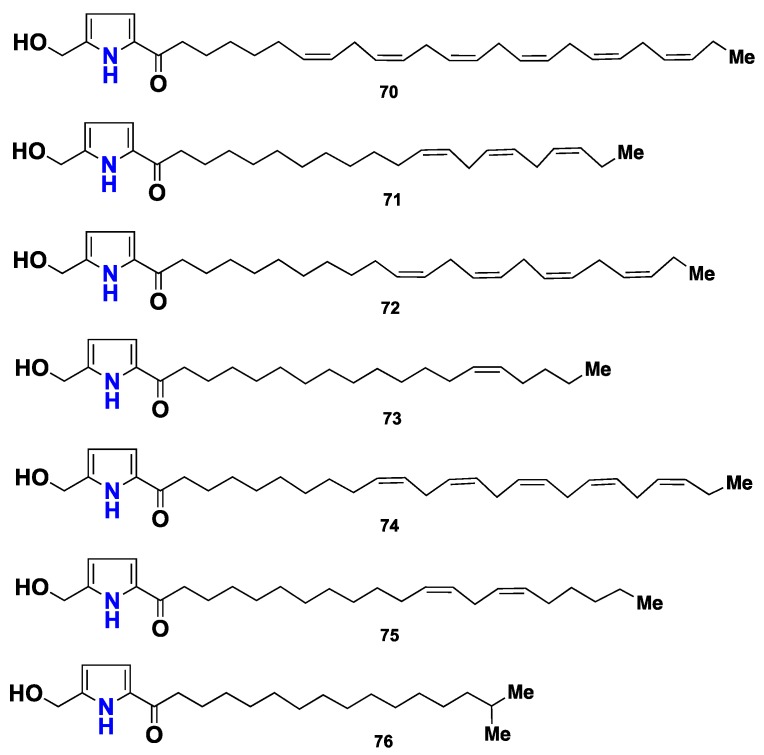
Chemical structures **70**–**76**.

**Figure 7:**
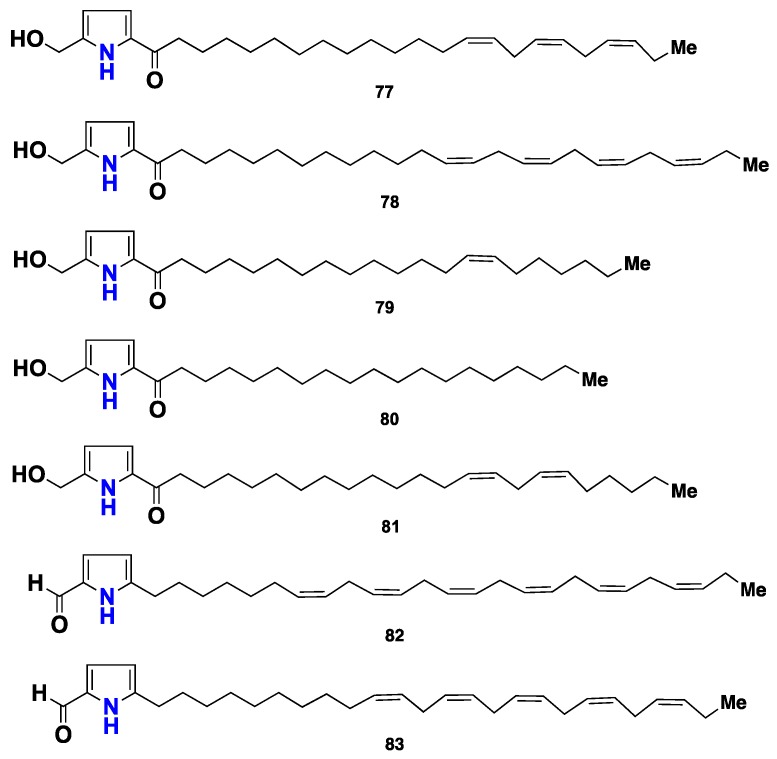
Chemical structures **77**–**83**.

Fourteen pyrrole-containing metabolites, named mycalazols (**70**–**81**) and mycalazals (**82**–**83**) (Figure 6 and Figure 7), were isolated from *M. micracanthoxea*, collected at the Southern coast of Spain. Compounds **70**–**83** displayed a potent in vitro cytotoxicity with ED_50_ values in the micromolar rang, against five cancer cell lines: P388, SCHABEL mice lymphoma, A549 human lung carcinoma, HT29 human colon carcinoma and MEL28 human melanoma, and **75**–**76** and **81** were the most active analogues [45].

**Figure 8:**
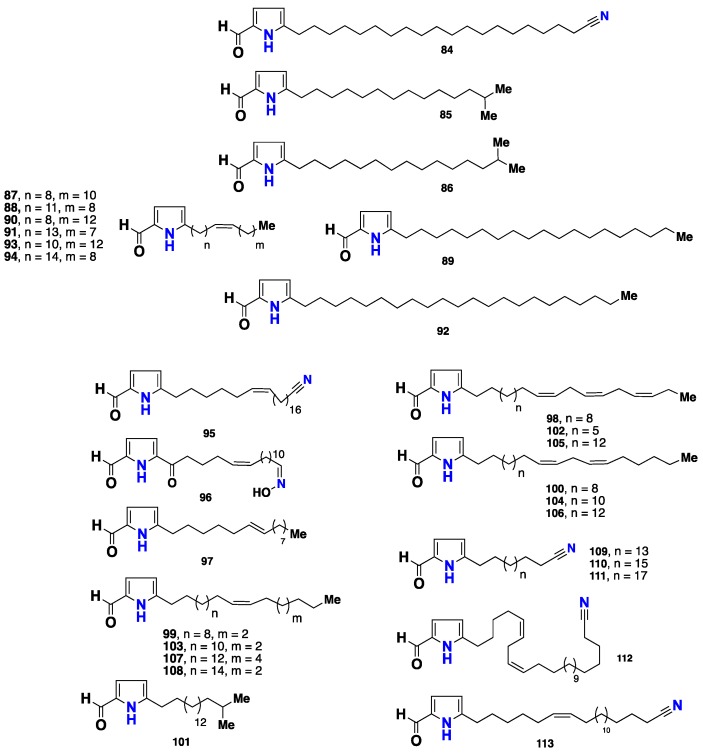
Chemical structures **84**–**113**.

A further eleven pyrrole-containing metabolites, **84**–**94** (Figure 8), were isolated from the same sponge, collected in the Caribbean Sea in Venezuela. The structures of these compounds were elucidated by analysis of their NMR, HRMS and GC-MS data. Compound **84** was the most active against *Leishmania mexicana* promastigotes, with LD_50_ value of 12 µg/mL [46]. Three 5-alkylpyrrole-2-carbaldehydes (**95**–**97**) (Figure 8) were reported from *M. tenuispiculata*, collected in Southern India [47], while an additional fourteen 5-alkylpyrrole-2-carbaldehyde analogues, with varying alkyl side chains, named mycalazals (**98**–**108**) and mycalenitriles (**109**–**111**) (Figure 8) were isolated from *M. Cecilia*, collected in California. These compounds displayed growth inhibition activity against nine cancer cell lines, with GI_50_ values below 5 µg/mL, being **103** the most cytotoxic against the LNcaP cell line, with a GI_50_ value of 0.2 µg/mL. Compounds **98**, **99** and **102** displayed remarkable cytostatic activity on this tumor cell line, with TGI (Total Growth Inhibition) values of 3.3, 2.6 and 2.8 µg/mL, respectively. Compounds **109**–**111** exhibited potent cytotoxicity with high selectivity against PANC1 human pancreas, LOVO human colon, and HELA human lymphoma cell lines [48]. It is interesting to point out that the cytotoxicity exhibited by mycalazals and mycalenitriles is affected by the structural features of the alkyl side chains, including their length, the number and position of the unsaturations [48]. Recently, Xue et al. [49] described isolation of mycalenitrile-15 (**112**) and mycalenitrile-16 (**113**) from the Chinese *M. lissochela*. Compound **112** displayed a remarkable PTP1B (Protein-tyrosine phosphatase 1B) inhibitory activity with an IC_50_ value of 8.6 µM.

### 2.5. Bromine-Containing Amides

**Figure 9:**
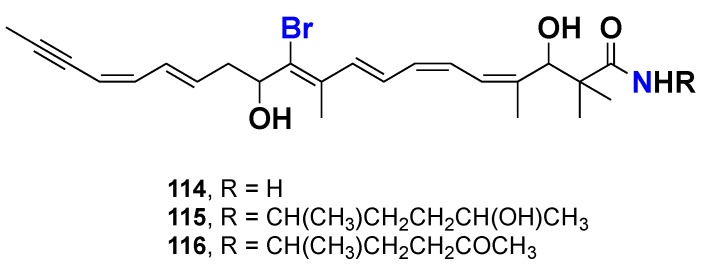
Chemical structures of **114**–**116.**

Three brominated acetylenic amides, clathrynamides A–C (**114**–**116**) (Figure 9), were isolated from the Japanese marine sponge *Clathria* sp., collected from the Sad-misaki coast. Compound **114** displayed potent inhibitory activity against the mitotic cell division of starfish eggs at a very low concentration, with an IC_50_ value of 6 ng/mL, and cytotoxicity against the human myeloid K-562 cell line with an IC_50_ value of 0.2 μg/mL. Compounds **115** and **116** were less active than **114** against the mitotic cell division of starfish eggs, with IC_50_ values of 0.2 and 1 μg/mL, respectively. Based on the IC_50_ values of **114**–**116**, it is clear that the presence of a primary amide in the molecule plays an important role in the inhibitory activity of the mitotic cell division of starfish eggs [50].

### 2.6. Cyclic Peptides/Thiopeptides

**Figure 10:**
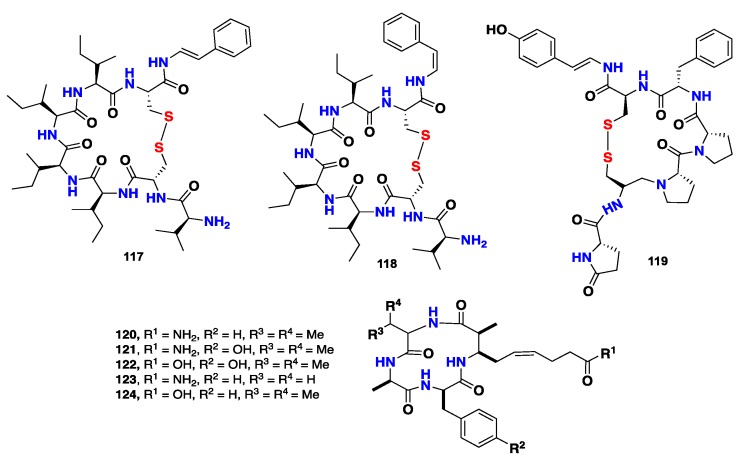
Chemical structures of **117**–**124.**

Two cyclic thiopeptides, microcionamides A (**117**) and B (**118**), were isolated from *C.* (*Thalysias*) *abietina*, collected from the Philippines. Compounds **117** and **118** displayed a significant cytotoxicity against the human breast tumor cell lines, MCF-7 and SKBR-3, with the IC_50_ values of 125/98 nM and 177/172 nM, respectively. Furthermore, **117** and **118** also displayed inhibitory activity against *Mycobacterium tuberculosis* (H_37_Ra), with MIC value of 5.7 μg/mL [51]. Another cyclic thiopeptide, gombamide A (**119**) (Figure 10) was isolated from the Korean marine sponge *C. gombawuiensis*. **119** exhibited a weak cytotoxicity against K562 and A549 cell lines with the IC_50_ values of 6.9 and 7.1 μg/mL, respectively. Moreover, **119** also exhibited a moderate inhibitory activity against Na^+^/K^+^-ATPase with IC_50_ of 17.8 μg/mL [52]. Five cyclic tetrapeptides, azumamides A–E (**120**–**124**), were isolated from the marine sponge *M. izuensis.* These compounds displayed a potent HDAC (Histone Deacetylase) inhibitory activity with the IC_50_ values of 0.045 to 1.3 µM, using enzymes obtained from K562 human leukemia cells. Compounds **120**–**124** represented the first examples of cyclic peptides with HDAC inhibition activity recorded from marine invertebrates [53].

### 2.7. Nucleotides

**Figure 11:**
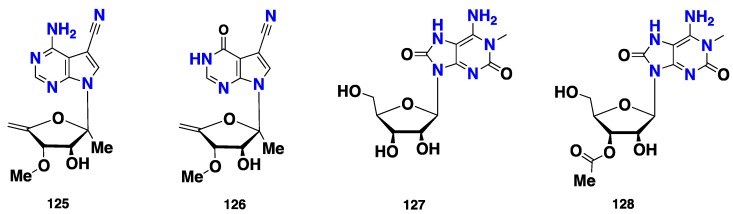
Chemical structures of **125**–**128**.

Two guanine-nucleotides, mycalisines A (**125**) and B (**126**) (Figure 11), from the Japanese sponge *Mycale* sp., were found to inhibit a cell division of the fertilized starfish (*Asterina pectinifera*) eggs with MIC_50_ of 0.5 and 200 µg/mL, respectively [54]. Two 8-oxoisoguanine-nucleotides, **127** and **128** (Figure 11), were isolated from *Clathria* (Microciona) *strepsitoxa*, collected from the Northeastern Atlantic. These compounds did not exhibit any significant antimicrobial or cytotoxic activity [55].

### 2.8. Fatty Acids

**Figure 12:**
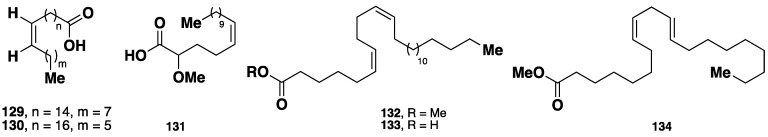
Chemical structures **129**–**134**.

(*Z*)-16-pentacosenoic acid (**129**) and (*Z*)-18-pentacosenoic acid (**130**) were isolated from the hydrolyzed phospholipids of the Caribbean sponge *M. laevis* [56], while (5*Z*)-2-methoxy-5-hexadeconic acid (**131**) (Figure 12) was reported from *M. laxissima* [57]. Chemical investigation of the Red Sea *M. euplectellioides* led to the identification of hexacosa-(6*Z*,10*Z*)-dienoic acid methyl ester (**132**), hexacosa-(6*Z*,10*Z*)-dienoic acid (**133**) and icosa-(8*Z*,11*Z*)-dienoic acid methyl ester (**134**) (Figure 12). Compounds **132**–**134** displayed weak cytotoxicity against A549 human lung carcinoma, U373 glioblastoma and PC-3 prostate cancer cell lines [58].

### 2.9. Polyketides Derivatives

**Figure 13:**
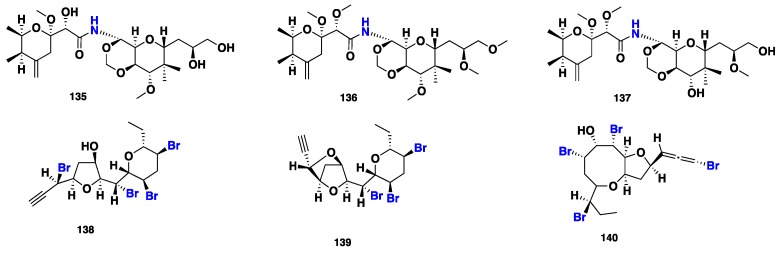
Chemical structures of **135**–**140**.

Mycalamides A (**135**) and B (**136**) (Figure 13) were isolated from *Mycale* sp., collected from Otago Harbour, New Zealand. Both compounds exhibited a potent in vitro anti-HSV-1 activity. Compound **136** was a more potent antiviral agent than **135**, with the Minimum Active Doses (MAD) of 1–2 and 3.5–5.0 ng/disk, respectively. Furthermore, **136** exhibited stronger (IC_50_ = 0.7 ± 0.3 ng/mL) cytotoxicity than **135** (IC_50_ = 3.0 ± 1.3 ng/mL) against P-388 cancer line [59,60]. Additionally, mycalamide D (**137**) (Figure 13), along with **135** and **136**, were also reported from *Mycale* sp., collected from New Zealand [61]. Compounds **135** and **137** displayed significant cytotoxicity against three cell lines: non-tumorigenic pig kidney (LLC-PK_1_), human lung carcinoma (H441) and human neuroblastoma (SH-SY5Y) cell lines. Furthermore, **135**–**137** exhibited remarkable cytotoxicity in a nanomolar range against lymphoma P-388 cells with IC_50_ values of 5.2, 1.3 and 65.5 ± 5.5 nM, respectively. From a structure-activity point of view, the cytotoxic potency is inversely proportional to the number of the methoxy groups as well as the polarity of the compounds (Figure 13) [61]. Within the polyketide group, acetogenins were also isolated from the marine sponge of the genus *Mycale*. Giordano et al. [62] reported the isolation of two polybrominated C_15_ acetogenins (**138**–**139**) from *M. rotalis*, and subsequently, Notaro et al. isolated the C_15_ nonrterpenoid brominated ether (**140**) from the same sponge [63].

### 2.10. Anthraquinones

**Figure 14:**
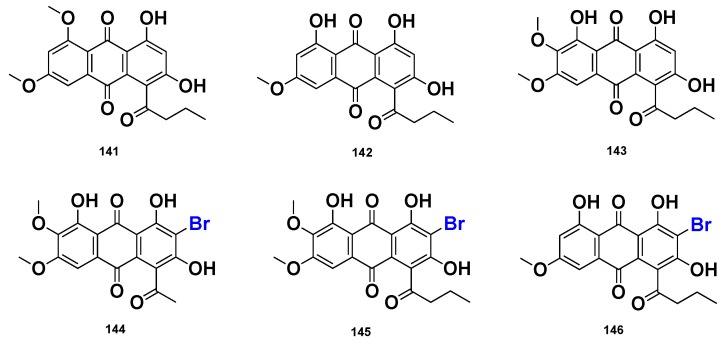
Chemical structures of **141**–**146**.

Six rhodocomatulin-type anthraquinones, including the previously reported rhodocomatulin 5, 7-dimethyl ether (**141**) and rhodocomatulin 7-methyl ether (**142**), together with the new 6-methoxyrhodocomatulin 7-methyl ether (**143**), 3-bromo-6-methoxy-12-deethylrhodocomatulin 7-methyl ether (**144**), 3-bromo-6-methoxyrhodocomatulin 7-methyl ether (**155**) and 3-bromorhodocomatulin 7-methyl ether (**146**) (Figure 14), were isolated from the marine sponge *C. hirsuta,* collected from the Great Barrier Reef, Australia. Compounds **141** and **142** were also isolated from the marine sponge *Comatula rotalaria* [64].

### 2.11. Macrolides

**Figure 15:**
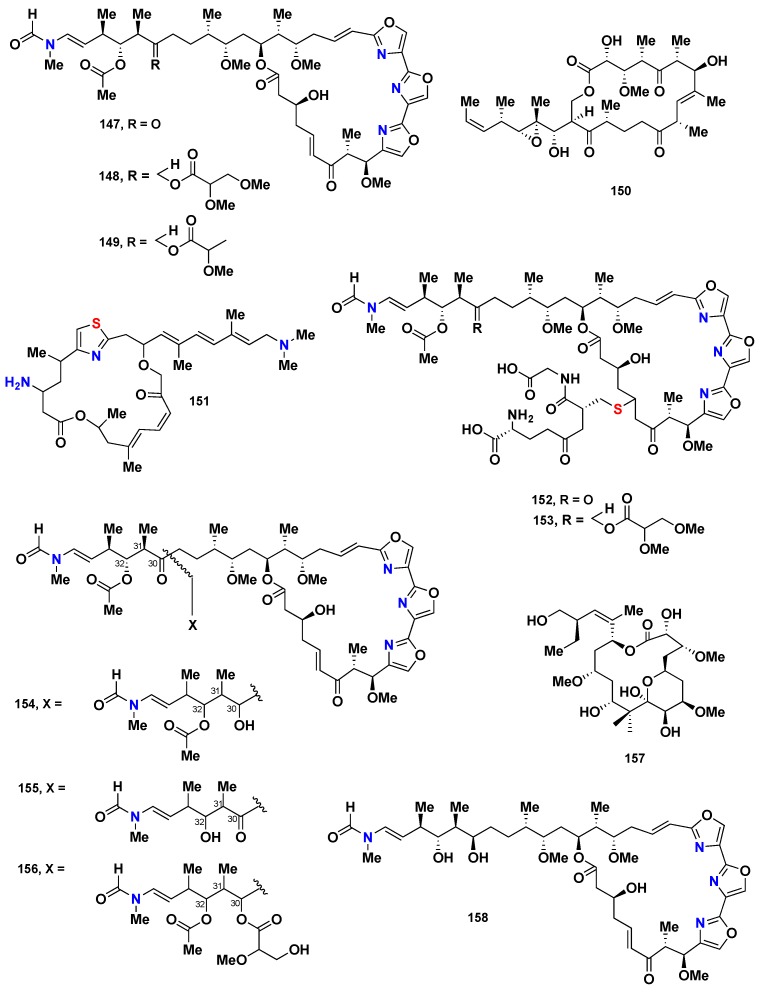
Chemical structures of **147**–**158**.

Three trioxazole containing macrolides, mycalolides A–C (**147**–**149**) (Figure 15), isolated from the Japanese *Mycale* sp., displayed antifungal activity against some pathogenic fungi. These compounds also showed a promising cytotoxicity against the B-16 cancer cell line, with IC_50_ values ranging from 0.5 to 1.0 ng/mL [65]. 13-Deoxytedanolide (**150**) (Figure 15), along with **147**–**148,** were also isolated from the Japanese sponge *M. adhaerens*. Compound **150** exhibited significant cytotoxicity against P388 leukemia cells, with an IC_50_ value of 94 pg/mL [66]. The chemical investigation of *Mycale* sp., collected from New Zealand, afforded a potent cytotoxic thiazole-containing macrolide, pateamine (**151**) (Figure 15). Compound **151** displayed significant and selective cytotoxicity against P388 cells with an IC_50_ value of 0.15 ng/mL [67]. Thiomycalolides A (**152**) and B (**153**) (Figure 15), another two trioxazole-containing macrolides, were reported from *Mycale* sp., collected at Gokasho Bay, Japan. Both compounds were cytotoxic against human leukaemia P388 cells with an IC_50_ of 18 ng/mL [68]. Further analogues, including 30-hydroxymycalolide A (**154**), 32-hydroxymycalolide A (**155**) and 38-hydroxymycalolide B (**156**) (Figure 15), were isolated, together with **147**–**149,** from the Japanese *M*. *magellanica*. Compounds **154**–**156** showed cytotoxicity against L1210 cells, with IC_50_ values of 0.019, 0.013 and 0.015 µg/mL, respectively [69,70]. Peloruside A (**157**), another cytotoxic macrolide, was isolated from *Mycale* sp., collected from New Zealand. This compound exhibited a remarkable cytotoxicity against P388 with IC_50_ value of 18 nM [71]. Additionally, 30, 32-dihydroxymycaloloide A (**158**) (Figure 15) was isolated from the Japanese sponge *M. izuensis* as a cytotoxic compound against HeLa cells with IC_50_ value of 2.6 ng/mL [72].

**Figure 16:**
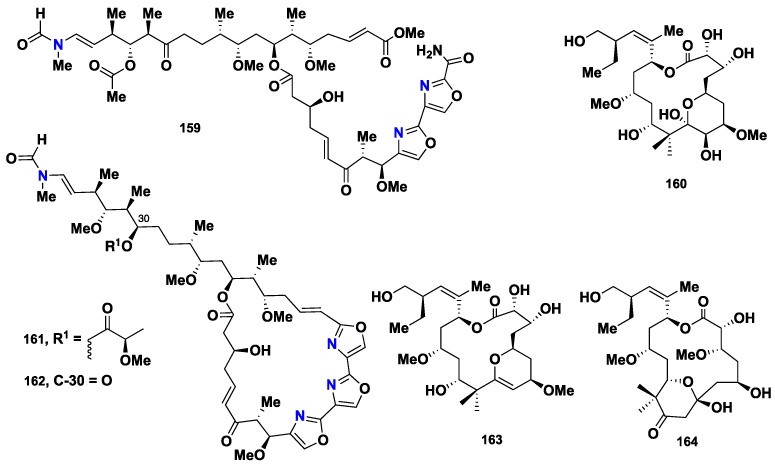
Chemical structures of **159**–**164**.

A bisoxazole-containing macrolide, secomycalolide A (**159**) (Figure 16)**,** was isolated from a Japanese *Mycale* sp., together with **147** and **154**. By using a chymotrypsin-like substrate, **159**, **147** and **154** displayed a promising proteasome inhibition activity, with IC_50_ values of 11, 30 and 45 µg/mL, respectively [73]. Later on, peloruside B (**160**) (Figure 16), another potent cytotoxic macrolide, was isolated from the New Zealand sponge *M. hentscheli*. Compound **160** showed strong cytotoxicity against human myeloid leukemia cells (HL-60) and human ovarian carcinoma 1A9 cells with IC_50_ values of 33 ± 10 and 71 ± 6 nM, respectively [74]. Additionally, miuramides A (**161**) and B (**162**) (Figure 16) were identified from *Mycale* sp., collected from Japan. Both compounds showed significant cytotoxicity against 3YI cells with IC_50_ value of 7 nM [75]. Very recently, Suo et al., described the isolation of pelorusides C (**163**) and D (**164**) (Figure 16), also from the New Zealand sponge *M. hentscheli.* Both compounds showed cytotoxicity against HL-60 cell line, with IC_50_ values of more than 2 and 15 µM, respectively [76]. A structure-activity analysis revealed that pelorusides A–D (**157**, **160**, **163** and **164**) (Figure 15 and Figure 16) stabilize microtubules by binding to β-tubulin, similar to the antitumor drug paclitaxel, highlighting the potential of these compounds as promising anticancer drug candidates [74,75,77,78,79]. 

### 2.12. Terpenoids

**Figure 17:**
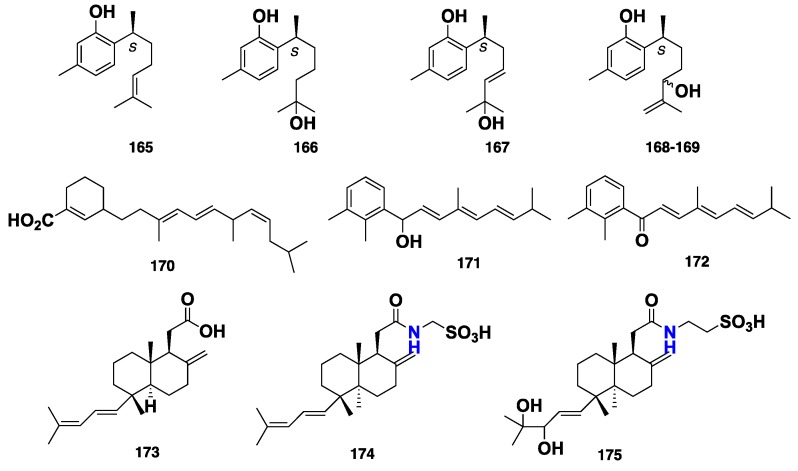
Chemical structures **165**–**175**.

**Figure 18:**
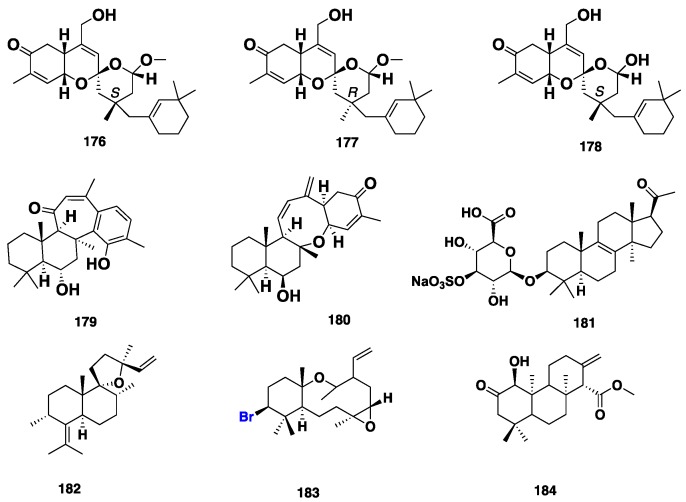
Chemical structures of **176**–**184**.

Five sesquiterpenes, including two sesquiterpene phenols (+)-curcuphenol (**165**) and (+)-curcudiol (**166**), along with three minor compounds, **167**–**169** (Figure 17), were reported from an Australian marine sponge *Mycale* (*Arenochalina*) sp. [80]. Compound **165** displayed in vitro cytotoxicity against P388 murine leukemia and human tumor cell lines (IC_50_ = 7 μg/mL), HCT-8 (colon; MIC = 0.1 μg/mL), mammary (MDAMB; MIC = 0.1 μg/mL) [80] and NSLC (A549; MIC = 10 μM) [81], as well as antifungal activity against *Candida albicans* and *Cryptococcus neoformans* (MIC = 15 μM) [81,82]. Moreover, **165** also showed antibacterial activity against both *Staphylococcus aureus* and methicillin-resistant *S. aureus*, with MIC value below 20 μM [82]. On the contrary, **166** only exhibited weak antifungal activity against filamentous fungi and *Candida albicans* with MIC = 250 μg/mL [81,83]. Three terpenoid metabolites, clathrins A–C (**170**–**172**) (Figure 17), were isolated from the marine sponge *Clathria* sp., collected from the Great Australian Bight. Compound **170** represents the first example of a marine sesquiterpene/benzenoid in which the “benzenoid” residue retained a nonaromatic shikimate character, while **171** and **172** are rearranged norditerpenes. However, **172** was thought to be an artefact, which represents the oxidized form of **171 [84]**. The unusual bicyclic C_21_-diterpenoids, including clathric acid (**173**) and two acyl taurine derivatives, clathrimides A (**174**) and B (**175**) (Figure 17), were isolated from the marine sponge *C. compressa*, which was collected in Florida [20]. These compounds were tested for antibacterial activity against several Gram-positive and Gram-negative bacteria. However, only **173** was found to exhibit weak antibacterial activity, with MIC = 32 μg/mL against *S. aureus* (ATTC 6538P)*,* and with MIC = 64 μg/mL against both methicillin-resistant *S. aureus* (ATTC 33591) and vancomycin-resistant *S. aureus* (VRSA), while **174** and **175** exhibited no activity at the highest concentration tested (128 μg/mL). Moreover, none of the compounds showed activity against Gram-negative bacteria [20]. Three tetracyclic sesterterpenes, gombaspiroketals A–C (**176**–**178**) (Figure 18), were isolated from the Korean sponge *C. gombawuiensis*, and showed in vitro cytotoxicity against K562 and A549 cell lines, with IC_50_ values of 1.45, 2.02, 0.85 and 0.77, 1.87, 4.65 μg/mL, respectively. Furthermore, **176** and **178** also exhibited antibacterial activity against several strains of Gram-positive bacteria, including *S. aureus*, *Bacillus subtilis* and *Kocuria rhizophila*, with MIC values of 25.0, 6.25, 12.5 and 25.0, 6.25, 25.0 μg/mL, respectively, and against Gram-negative bacteria *Salmonela enterica* and *Proteus hauseri*, with MIC values of 12.5, 6.25 and 25.0, 12.5 μg/mL, respectively. Moreover, **176**–**178** also inhibited the enzymes Na^+^/K^+^-ATPase and isocitrate lyase (ICL) with IC_50_ = 10.9, 77.9, 18.7 and 57.4, >100, 66.3 μg/mL, respectively, and their inhibitory activity was hypothesized to be due to the three-dimensional structure of the spiroketal motif [85]. Phorone B (**179**) and ansellone C (**180**) (Figure 18), along with a nortriterpene sodium *O-*sulfonato-glucuronide saponin gombaside A (**181**) (Figure 18), were also isolated from *C. gombawuiensis*. Compound **181** features a rare 4,4,14-trimethyl pregnane skeleton. Compounds **179**–**181** exhibited moderate cytotoxicity against A549 and k562 cancer cell lines with IC_50_ values of 4.7/3.9, 5.4/4.5 and 2.1/1.8 μg/mL, respectively. Interestingly, while **181** showed antibacterial activity against *B. subtilis* and *P. hauseri* with MIC values of 1.6 and 3.1 μg/mL, respectively, **179** and **180** were inactive (MIC > 100 μg/mL) [86]. Rotalins A (**182**) and B (**183**) are two diterpenes reported from the Mediterranean *M. rotalis* [87] while mycgranol (**184**) (Figure 18) is a diterpene, isolated from the Kenyan *M*. aff. *graveleyi* [88].

**Figure 19:**
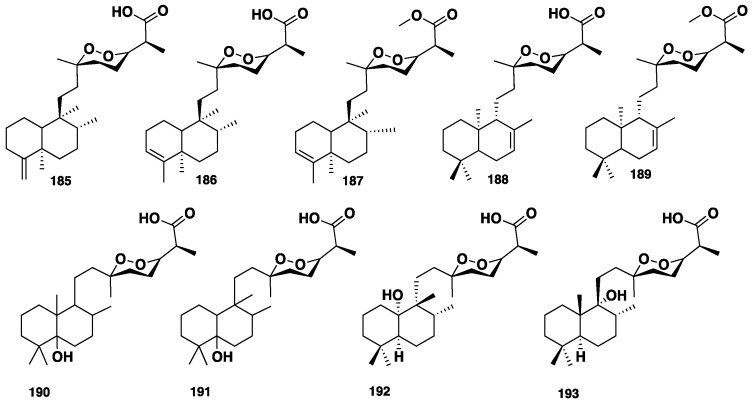
Chemical structures **185**–**193**.

**Figure 20:**
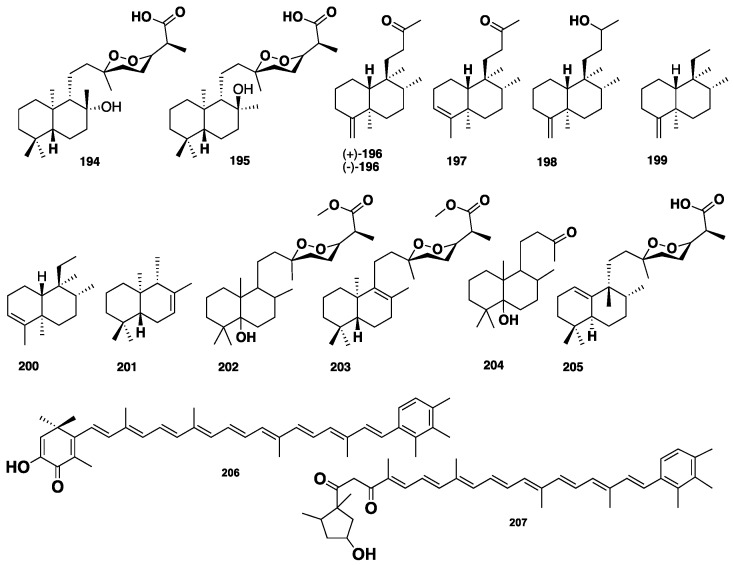
Chemical structures of **194**–**207**.

Norsesterterpene cyclic peroxides are a distinct class of marine sponges-derived metabolites. Five norsesterterpene cyclic peroxides, **185**–**189** (Figure 19), were isolated from the Australian marine sponge *M. ancorina* [89]*.* Capon et al. [90] reported the isolation of further two norsesterterpene cyclic peroxides, **190**–**191** (Figure 19), from *M.* (*Carmia*) cf. *spongiosa*, collected from New South Wales, Australia. Compounds **190**–**191** were isolated from the CH_2_Cl_2_ soluble fraction, which exhibited antimicrobial activity against *B. subtilis* and *Saccharomyces cerevisae*. Mycaperoxides A (**192**) and B (**193**) (Figure 19), isolated from the Thai *Mycale* sp., were found to display in vitro potent cytotoxicity against three cancer cell lines, P-388, A-549 and HT.29 with IC_50_ of 0.5-1.0 µg/mL, and antiviral activity against several viruses, including HSV-1. Moreover, these compounds also showed antibacterial activity against *B. subtilis and S. aureus* [91]. A further five cyclic peroxides, **185**, **186**, **189**, mycaperoxides C (**194**) and D (**195**), along with six norterpenes, **196**–**201** (Figure 20), were isolated from *M.* sp. From Australia [92]. A re-investigation of *Mycale* sp., collected from New South Wales, Australia, allowed the identification of two further mycaperoxides F (**202**) and G (**203**) and a norterpene ketone (**204**) (Figure 20) [93]. Similarly, re-examination of the Thai *Mycale* sp., by Phuwapraisirisan et al., led to the isolation of mycaperoxide H (**205**) (Figure 20), which was cytotoxic against the HeLa cancer cell line with IC_50_ = 0.8 µg/mL [94].

Two aromatic keto-carotenoids, clathriacine (**206**) and trikentriohodine (**207**) (Figure 20), were isolated from the marine sponge identified as *C. frondifera*, which, if correct, is now a junior synonym of *C.* (*Thalysias*) *vulpine* [95,96].

### 2.13. Steroidal Compounds

**Figure 21:**
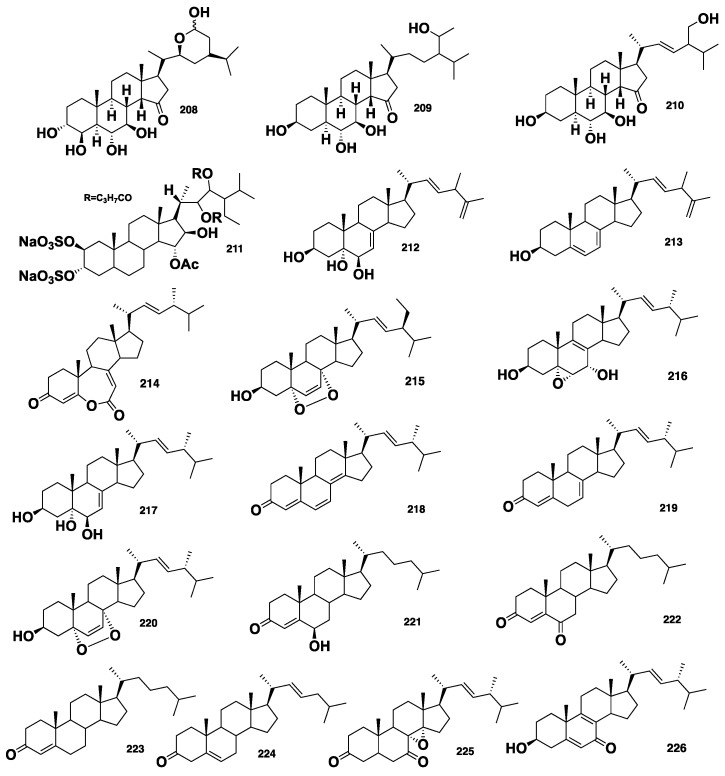
Chemical structures of **208**–**226**.

**Figure 22:**
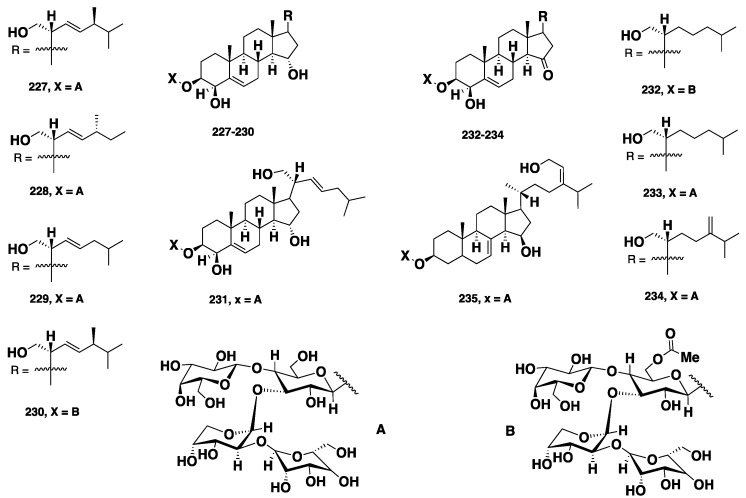
Chemical structures **227**–**235**.

Three highly oxygenated steroids, named contignasterol (**208**) and clathriols A (**209**) and B (**210**) (Figure 21), were isolated from the New Zealand marine sponge *C.* (*Clathria*) *lissosclera.* While **208** exhibited a histamine release inhibitory activity with an IC_50_ = 0.8 ± 0.32 μg/mL, **209** and **210** showed anti-inflammatory activity against the production of superoxide stimulated with *N*-formyl-methionine-leucine-phenylalanine (fmlp) or phorbol myristate acetate (PMA), with IC_50_ values of −33/27 and 140/130 μg/mL, respectively. Moreover, **209** also displayed a 72% inhibition of the histamine release in peritoneal mast cells and a 76% inhibition of human peripheral blood neutrophil at a concentration of 30 μM [17,18]. Bioassay-guided fractionation of the CHCl_3_-MeOH crude extract of the marine sponge *Clathria* sp., collected from the Red sea, resulted in the isolation of a sulfated sterol, clathsterol (**211**) (Figure 21), which displayed moderate antiviral activity against HIV-1 at a concentration of 10 μg/mL [16]. Biemansterol (**212**), along with the previously reported 24β-methylcholesta-5, 7, 22, 25-tetraen-3β-ol (**213**) (Figure 21), were isolated from the Okinawan marine sponge *Biemna* sp. [97]. Compound **212**, which possesses a rare 22, 25-diene side chain displayed in vitro cytotoxicity against murine lymphoma L1210 and human epidermoid KB cell lines, with IC_50_ values of 3 and 1.3 μM, respectively [97]. Foristerol (**214**) (Figure 21), a steroid featuring an unusual seven-membered lactone ring, was reported from the Chinese marine sponge, *B. fortis* [98], while 5α, 8α-epidioxy-24(*S*)-ethylcholest-6-en-3β-ol (**215**) (Figure 21) was isolated from the Madagascar marine sponge *B. triraphis* [99]. Huang and Guo [100] described the isolation of nine steroids, including melithasterol B (**216**), (24*R*)-ergosta-7,22-dien-3, 5, 6-triol (**217**), (24*R*)-ergosta-4, 6, 8(14), 22-tetraen-3-one (**218**), (24*R*)-ergosta-4, 7, 22-trien-3-one (**219**), (24*R*)-ergosta-6, 22-dien-5, 8-epidioxy-3-ol (**220**), 6-hydroxycholest-4-en-3-one (**221**), cholest-4-en-3, 6-dione (**222**), cholest-4-en-3-one (**223**) and cholest-5, 22-dien-3-one (**224**) (Figure 21), from the Chinese marine sponge *B. fortis*. Compound **222** displayed mild inhibition of T- and B-lymphocytes proliferation and potent hPTP1B inhibitory activity, with IC_50_ = 1.6 μM [100]. Youssef et al. [101] reported the isolation of ehrenasterol (**225**) and (22*E*)-ergosta-5, 8, 22-trien-7-one-3β-ol (**226**), along with the previously reported (24*R*)-ergosta-6, 22-dien-5, 8-epidioxy-3-ol (**220**) and **216** (Figure 21), from the Red Sea marine sponge *B. ehrenbergi*. Compound **225** exhibited antibacterial activity with an inhibition zone of 20 mm at 100 μg/disc against *E. coli*. Moreover, both **225** and **226** showed weak cytotoxicity against a human colon adenocarcinoma (HCT-116) cancer cell line, with IC_50_ of 45 and 40 μg/mL, respectively [101]*.* The steroidal oligoglycosides, mycalosides A–I (**227**–**235**) (Figure 22), were isolated from the Caribbean sponge *M*. *laxissima.* These compounds represent the first examples of steroidal oligoglycosides reported from marine sponges. The fraction containing **227**–**235**, along with the pure mycaloside A (**227**) and mycaloside G (**233**), showed growth inhibition of fertilized eggs of the marine urchin (*Strongylocentrotus nudus*), with EC_50_ of 7.4 and 3.2 µg/mL, respectively [102,103].

### 2.14. Miscellaneous Compounds

**Figure 23:**
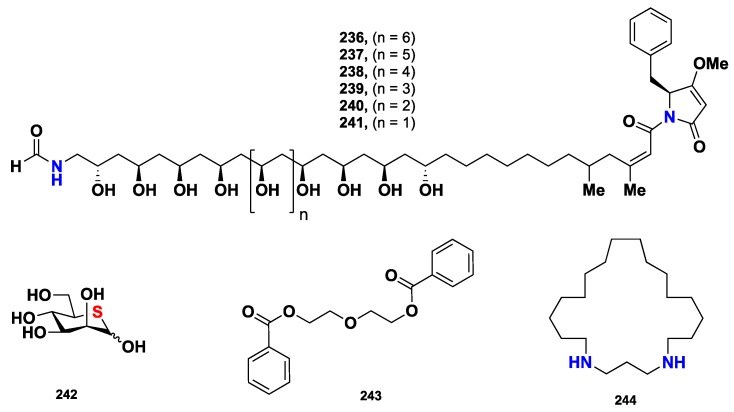
Chemical structures **236**–**244.**

The unprecedented cytotoxic PKS/NPRS metabolites, mycapolyols A–F (**236**–**241**) (Figure 23), were isolated from a Japanese *M. izuensis*. These compounds displayed cytotoxic activity against the HeLa cells, with IC_50_ values of 0.06, 0.05, 0.16, 0.40, 0.38 and 0.90 µg/mL, respectively [104]. On the other hand, the first naturally occurring 5-thiosugar, 5-thio-D-mannose (**242**) (Figure 23), was reported from the marine sponge *C.* (*Dendrocia*) *pyramida* [105] while diethylene glycol dibenzoate (**243**) was reported from *C. reinwardtti*, collected at the Mandapam coast in the Gulf of Mannar, Tamilnadu, India [106]. 1,5-Diazacyclohenicosane (**244**) (Figure 23), an aliphatic cyclic diamine was isolated from the Kenyan *Mycale* sp. [107]. Compound **244** exhibited significant cytotoxicity against A549 human lung carcinoma, HT29 human tissue carcinoma, and MDA-MB-231 human breast adenocarcinoma, with GI_50_ values of 5.41, 5.07 and 5.74 µM, respectively.

## 3. Conclusions and Prospects

This review presents extensive documented data, focusing on chemical diversity and biological activities of the secondary metabolites, isolated from the three marine sponge genera: *Mycale* (*Arenochalina*)*, Biemna* and *Clathria*, demonstrating these marine species as prolific sources of structurally diverse bioactive compounds. Despite their production of tricyclic guanidine-containing alkaloids, these sponges are classified under two different orders: *Mycale* (*Arenochalina*)*/Clathria* (under the order Poecilosclerida) and *Biemna* (under the order Biemnida), as recent molecular data revealed that *Biemna* is not related to the Poecilosclerida, and hence a new order Biemnida was given for the genus *Biemna*. This finding could highlight the important question of using secondary metabolites as taxonomic markers. Another important chemical feature is the uniqueness of the production of pyridoacridine alkaloids by *Biemna* sponges, which implies the relatedness of *Biemna* genus to the order Poecilosclerida. The two hundred and forty-four metabolites reported in this review are put together into fourteen major chemical classes, according to their structural characteristics and biosynthetic origin. The vast array of bioactivities exhibited by some of these metabolites make these marine sponge genera some of the most attractive biological targets, worthy of further exploration.

## Figures and Tables

**Table 1:** Summary of the secondary metabolites isolated from the marine sponges belonging to the genera *Mycale* (*Arenochalina*), *Biemna* and *Clathria*, their source organisms and biological activities.

Name	Compound Class	Marine Sponges	Collection	Bioactivities	Ref.
Crambescidin 800 (**1**)	Pentacyclic guanidine	*Clathria* (*Thalysias*) *cervicornis*	-	Antimicrobial	21
Crambescidins **1**–**6**	Pentacyclic guanidine	*C. bulbotoxa*	Indonesia	Cytotoxic, antifungal	28
Norbatzelladine L (**7**)	Tricyclic guanidine	*C.* (*Microciona*) *calla*	Caribbean	Cytotoxic	29
Clathriadic acid (**8**)	Tricyclic guanidine	*C.* (*Microciona*) *calla*	Caribbean	Cytotoxic, antimalarial	29
Mirabilins A–F (**9**–**14**)	Tricyclic guanidine	*Mycale* (*Arenochalina*) *mirabilis*	Australia	Nr	30
Netamines A–G (**15**–**21**)	Tricyclic guanidine	*Biemna laboutei*	Madagascar	Cytotoxicity	31
Netamines H–N (**22**–**28**)	Tricyclic guanidine	*B. laboutei*	Madagascar	Cytotoxic, antimalarial	14
Netamines O–S (**29**–**33**)	Tricyclic guanidine	*B.laboutei*	Madagascar	Cytotoxic, antimalarial	15
Mirabilin G (**34**)	Tricyclic guanidine	*Clathria* sp.	Australia	Antibacterial, antifungal	32
Mirabilins H–J (**35**–**37**)	Tricyclic guanidine	*Clathria* sp.	Australia	Cytotoxic	33
Araiosamines A–D (**38**–**41**)	Indole cyclic guanidine	*C.* (*Thalysias*) *araiosa*	Vanuatu	Antibacterial, Anti-HIV-1	34
**42**–**45**	Pyridoacridine	*Biemna* sp.	Okinawa	Cytotoxicity	37
**46** and **47**	Pyridoacridine	*Biemna* sp.	Indonesia	Enzyme inhibitor	38
**48** and **49**	Pyridoacridine	*Biemna* sp.	Japan	Cytotoxic	39
**50**–**53**	Pyridoacridine	*Biemna* sp.	Japan	Cytotoxic	40
Pseudoanchnazines A–C (**54**–**56**)	Pteridine alkaloid	*Clathria* sp.	Argentina	Antibacterial	41
Clathryimine A (**57**)	Quinolizine alkaloid	*C.* (*Clathria*) *basilana*	Indo-Pacific	Nr	42
*N*-methylpyrrolidone (**58**)	Pyrrolodine Alkaloid	*C. frondifera*	India	Nr	43
**59**–**69**	Indole alkaloids	*M. fibrexilis*	China	Nr	44
**70**–**83**	Pyrrole alkaloids	*M. micracanthoxea*	Spain	Cytotoxic	45
**84**–**94**	Pyrrole alkaloids	*M. micracanthoxea*	Venezuela	Cytotoxic	46
**95**–**97**	Pyrrole alkaloids	*M. tenuispiculata*	India	Nr	47
**98**–**111**	Pyrrole alkaloids	*M. cecilia*	California	Cytotoxic	48
**112** and **113**	Pyrrole alkaloids	*M. lissochela*	China	Enzyme inhibitor	49
Clathrynamides A–C (**114**–**116**)	Bromine-containing amide	*Clathria* sp.	Sad-Misaki, Japan	Cytotoxic, inhibitors of starfish eggs	50
Microcionamides A&B (**117**&**118**)	Cyclic thiopeptide	*C.* (*Thalysias*) *abietina*	Philippines	Cytotoxic, antibacterial	51
Gombamide A (**119**)	Cyclic thiopeptide	*C.* (*Clathria*) *gombawuiensis*	Korea	Cytotoxic, enzyme inhibitor	52
Azumamides (**120**–**124**)	Cyclic peptides	*Mycale izuensis*	Japan	Histone Deacetylase	53
Mycalisines (**125**–**126**)	Nucleotides	*Mycale* sp.	Japan	Inhibitors of starfish eggs	54
**127** and **128**	Nucleotides	*C*. (*Microciona*) *strepsitoxa*	Atlantic	Nr	55
**129** and **130**	Fatty acid	*M. laevis*	Caribbean	Nr	56
**131**	Fatty acid	*M. laxissima*	Caribbean	Nr	57
**132**–**134**	Fatty acid	*M. euplectellioides*	Red Sea	Cytotoxic	58
Mycalamides A&B (**135**&**136**)	Polyketide	*Mycale* sp.	New Zealand	Cytotoxic, antiviral	59–60
Mycalamide D (**137**)	Polyketide	*Mycale* sp.	New Zealand	Cytotoxic	61
**138**–**140**	Polyketide	*M. rotalis*	Mediterranean	Nr	62–63
**141**–**146**	Anthraquinone	*C.* (*Thalysias*) *hirsuta*	Australia	Nr	64
**147**–**149**	Macrolide	*Mycale* sp.	Japan	Antifungal, cytotoxic	65
**150**	Macrolide	*M. adhaerens* Lamb	Japan	Cytotoxic	66
Pateamine (**151**)	Macrolide	*Mycale* sp.	New Zealand	Cytotoxic	67
**152** and **153**	Macrolide	*Mycale* sp.	Japan	Cytotoxic	68
**154**–**156**	Macrolide	*M. magellanica*	Japan	Cytotoxic	69–70
Peloruside A (**157**)	Macrolide	*Mycale* sp.	New Zealand	Cytotoxic	71
**158**	Macrolide	*M. izuensis*	Japan	Cytotoxic	72
**159**	Macrolide	*Mycale* sp.	Japan	Cytotoxic	73
Peloruside B (**160**)	Macrolide	*M. hentscheli*	New Zealand	Cytotoxic	74
**161** and **162**	Macrolide	*Mycale* sp.	Japan	Cytotoxic	75
Peloruside C&D (**163**&**164**)	Macrolide	*M. hentscheli*	New Zealand	Cytotoxic	76
**165-169**	Sesquiterpene	*M.* (*Arenochalina*) sp	Australia	Antitumor, antifungal	80–83
Clathrin A–C (**170**–**172**)	Sesterterpene	*Clathria* sp	Australia	-	84
Clathric acid (**173**)	C_21_ terpenoid	*C. compressa*	Florida	Antimicrobial	20
Clathrimide A&B (**174**&**175**)	C_21_ -terpenoid	*C. compressa*	Florida	Antimicrobial	20
Gombaspiroketal A–C (**176**–**178**)	Sesterterpene	*C. gombawuiensis*	Korea	Antibacterial, enzyme inhibitors	85
**179** and **181**	Norterpene/triterpene	*C. gombawuiensis*	Korea	Antibacterial	86
Rotalins (**182**–**183**)	Diterepene	*M. rotalis*	Mediterranean	Nr	87
Mycgranol (**184**)	Diterepene	*M*. aff. *graveleyi*	Kenya	Nr	88
**185**–**189**	Cyclic norterpenoid peroxide	*M. ancorina*	Australia	Nr	89
**190** and **191**	Cyclic norterpenoid peroxide	*M.* (carmia) cf. *spongiosa*	Australia	Antimicrobial	90
**192** and **193**	Cyclic norterpenoid peroxide	*Mycale* sp.	Thailand	Cytotoxic, antiviral	91
**194**–**201**	Cyclic peroxide/norditerepene	*Mycale* sp.	Australia	Nr	92
**202**–**204**	Cyclic norterpenoid peroxide	*Mycale* sp.	Australia	Nr	93
**205**	Cyclic norterpenoid peroxide	*Mycale* sp.	Thailand	Cytotoxic	94
**206** and **207**	Tetraterpene	*C. frondifera* (*=C.* (*Thalysias vulpina*)	Japan	Nr	95–96
Contignasterol (**208**)	Steroid	*C.* (*Clathria*) *lissosclera*	New Zealand	Histamine inhibitory	17–18
Clathriols A&B (**209**&**210**)	Steroid	*C.* (*Clathria*) *lissosclera*	New Zealand	Anti-inflammatory, histamine inhibitory	17–18
Clathsterol (**211**)	Sulphated sterol	*Clathria* sp.	Red Sea	Anti-HIV-1	16
Biemansterol (**212**)	Sterol	*Biemna* sp.	Okinawa, Japan	Cytotoxic	97
**213**	Sterol	*Biemna* sp.	Okinawa, Japan	Cytotoxic	97
Foristerol (**214**)	Sterol	*B. fortis*	China	Nr	98
**215**	Sterol	*B. triraphis*	Madagascar	Nr	99
**216**–**224**	Sterol	*B. fortis*	China	Lymphocytes and hPTP1B inhibition	100
**225** and **226**	Sterol	*B. ehrenbergi*	Red Sea	Cytotoxic, antibacterial	101
**227**–**235**	Sterol	*M*. *laxissima*	Caribbean	Fertilized eggs inhibitors	102–103
Mycapolyols A–F (**184**–**189**)	Mixed PKS/NPRS	*M. izuensis*	Japan	Cytotoxic	104
2**42**	Thio-sugar	*C.* (*Dendrocia*) *pyramida*	Australia	Nr	105
**243**	Glycol	*C.* *reinwardtti*	India	Nr	106
**244**	1,5-Diamine	*Mycale* sp.	Kenya	Cytotoxic	107

Nr: Not recorded.

## Abbreviations

The following abbreviations are used in this manuscript:

EC_50_	Half maximal Effective Concentration
GI_50_	Half maximal Growth Inhibition
HIV-1	Human Immunodeficiency Virus 1
IC_50_	Half maximal Inhibitory Concentration
MIC	Minimum Inhibitory Concentration
MAD	Minimum active Dose
MID	Minimal Infective Dose
LD_50_	Lethal Dose 50 (median concentration of a toxicant that will kill 50% of the test animals within a designated period
